# The Tree of Life, Archetype and Artifice

**DOI:** 10.3201/eid3008.AC3008

**Published:** 2024-08

**Authors:** Byron Breedlove

**Affiliations:** Centers for Disease Control and Prevention, Atlanta, Georgia, USA

**Keywords:** Gustav Klimt, The Tree of Life, Stoclet Frieze, bacteria, archaea, Eukaryota, diversity of life, microbiome, art–science connection, horizonal gene transfer

**Figure 1:**
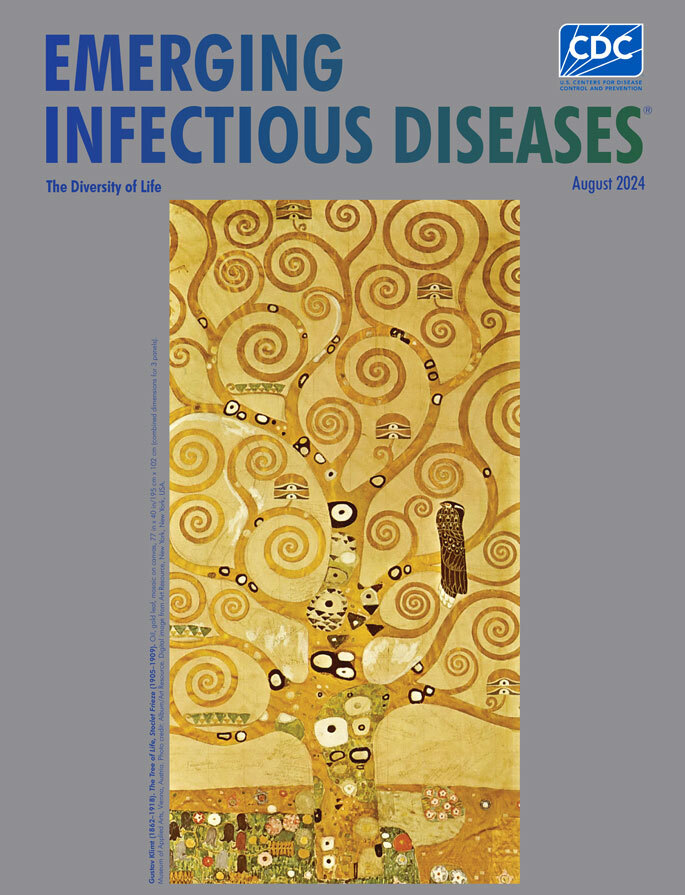
**Gustav Klimt** (1862–1918). *The Tree of Life, Stoclet Frieze,* (1905–1909). Oil, gold leaf, mosaic on canvas, 77 in x 40 in/195 cm x 102 cm (combined dimensions for 3 panels). Museum of Applied Arts, Vienna, Austria. Photo credit: Album/Art Resource. Digital image from Art Resource, New York, New York, USA.

The tree of life is an archetype woven into various mythological, religious, philosophical, and shamanistic traditions around the world, as well as a concept underpinning some scientific efforts to depict the diversity of life. It is also a tantalizing subject for visual artists, and this month’s cover image features an example, *The Tree of Life,* from the *Stoclet Frieze* by Gustav Klimt.

Klimt was born in 1862 in Baumgarten, Austria, then a small village near Vienna. His artistic talents were apparent from a young age, and when just 14 years old, he attended the Vienna School of Arts and Crafts on a scholarship. His brother Ernst later joined him, and they focused on becoming architectural painters. After graduation in 1883, the Klimt brothers and fellow artist Franz Matsch formed a successful studio, named Künstler-Compagnie (Artists’ Company), which received commissions for creating murals and art for public spaces such as theaters and museums. An uncredited online biography of Klimt recounts, “Their most notable works during this time were the mural at the Vienna Burgtheater and the ceiling above the staircase at the Kunsthistorisches Museum. The group was honored for their achievements in 1888, when they received the Golden Order of Merit from Austro-Hungarian Emperor Franz Josef I.”

After Gustav Klimt’s father and brother, both named Ernst, both died in 1892, Klimt shifted to more personal and less traditional artistic pursuits. In 1897, he was among the Austrian artists who formed the Vienna Secession, a movement that favored symbolism and allegory over realism. Art historian Dusan Nikolic wrote, “Under the presidency of Klimt, they founded the Union of Austrian Artists Vienna Secession, intending to educate society through future-oriented artistic concepts and infusing life with art.” Rifts among this group began, and Nikolic noted that Klimt, “whose style became a combination of natural elements with large areas of abstract geometrical ornament,” and other prominent members who also favored abstract style over realism left the movement in 1908.

**Figure 2:**
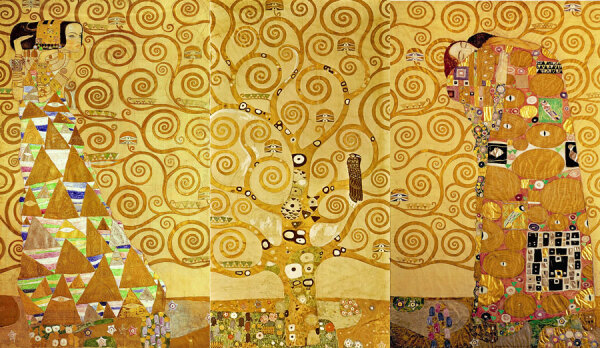
*Stoclet Frieze* by Gustav Klimt, depicting a standing female figure, a central panel known as *The Tree of Life*, and a couple embracing.

The *Stoclet Frieze* is among the best-known works from Klimt’s “Golden Phase,” which started a few years before he broke from the Vienna Secession. A wealthy Belgian couple, Adolphe and Suzanne Stoclet, commissioned Klimt to design a series of mosaics for the dining room at their Palais Stoclet in Brussels. Klimt’s panels depict a standing female figure, a central panel known as *The Tree of Life*, and a couple embracing (Figure 2). Klimt worked on that intricate ornamental mosaic for 5 years, incorporating semiprecious stones, gilded tiles, enamel, marble, ceramics, gold leaf, and other opulent materials. Art historian A.N. Hodge noted that it showcases Klimt’s “mastery of mosaic, a technique he had intensely studied on his recent trip to Ravenna [Italy].”

The center panel features a mosaic of a golden tree with curling, spiraling branches, festooned with recurring decorative shapes. The tree grows from an ochre patch of earth, and dazzling jeweled flowers cluster around the trunk. Klimt’s opulent and luminescent tree celebrates the continuity and complexity of life. Perched on a lower branch is a large black bird that contrasts with the brightly shimmering gold background, perhaps intended by Klimt as a symbol of death.

Klimt’s tree is symbolic, but in the biological sciences, the tree motif proved, for a time, a useful working artifice. Charles Darwin wrote, “The affinities of all the beings of the same class have sometimes been represented by a great tree. I believe this simile largely speaks the truth.” Science writer David Quammen explores this topic in his book *The Tangled Tree: A Radical New History of Life* and documents how trees have been used to categorize the diversity of life forms that emerged and evolved from some common ancestral microbe billions of years ago. Quammen discusses trees sketched by Charles Darwin and Ernst Haeckel in the 17th Century, provides example of trees from the 18th- and 19th-Century scientific literature, and shows modern iterations created by 20th-Century scientists.

Modern evolutionary classification is a rapidly changing science, especially regarding microbial life. In 1977, a team of researchers lead by biophysicist Carl Woese announced their discovery of a new, distinct category of microbial life now known as Archaea. Woese’s work made previous trees of life obsolete and new depictions of such trees, including his own, more contorted. Scientists now categorize life on earth into 3 domains: Bacteria, Archaea, and Eukaryota. Those domains are further subdivided into 6 kingdoms: Animalia, Plantae, Fungi, Protista, Archaebacteria, and Eubacteria.

Mapping life’s diversity via the tree motif was further complicated after the 20th-Century discovery of horizonal gene transfer—that is, the movement of DNA between organisms instead of transmission from parent to offspring. Such sideways transfer occurs across species and across kingdoms, blurring those boundaries, and contributes to the spread of antimicrobial resistance. Quammen notes that “the tree hypothesis works poorly for the history of bacteria and archaea, with all their sideways exchanges; and it works imperfectly for everything else.”

This month’s issue of *Emerging Infectious Diseases* focuses on the diversity of life, and an article by Stefanie Duller and Christine Moissl-Eichinger examines how archaea in the human microbiome are linked to various diseases, “though archaea are generally considered nonpathogenic.” Other articles discuss a range of emerging infections from various kingdoms, including different bacterial, fungal, and viral agents.

In his 1999 article Phylogenetic Classification and the Universal Tree, biologist W. Ford Doolittle stated, “Molecular phylogeneticists will have failed to find the ‘true tree,’ not because their methods are inadequate or because they have chosen the wrong genes, but because the history of life cannot properly be represented as a tree. However, taxonomies based on molecular sequences will remain indispensable, and understanding of the evolutionary process will ultimately be enriched, not impoverished.” Even if the tree motif does not serve as an accurate artifice to illustrate the diversity of life, especially at the microbial level, Klimt’s *The Tree of Life* resonates as a celebration of this archetypal image.
